# Subarachnoid Fluid Lactate and Paraplegia after Descending Aorta Aneurysmectomy: Two Compared Case Reports

**DOI:** 10.1155/2013/925739

**Published:** 2013-10-03

**Authors:** Enrico Giustiniano, Silvia Eleonora Malossini, Francesco Pellegrino, Franco Cancellieri

**Affiliations:** Department of Anesthesia and Intensive Care Unit, Humanitas Clinical and Research Center, Via Manzoni 56, 20089 Rozzano, Italy

## Abstract

We report a comparison of two cases regarding subjects who underwent thoracoabdominal aorta aneurysmectomy. During the procedure we monitored cerebrospinal fluid lactate concentration. One patient experienced postoperative paraplegia and his cerebrospinal fluid lactate concentration was much higher than that in the other case, whose postoperative outcome was uneventful. Consequently we consider that monitoring the lactate concentration in cerebrospinal fluid during thoracic aorta surgical procedures may be a helpful tool to predict the ischemic spine-cord injury allowing for trying to recover it precociously.

## 1. Introduction

During descending aorta surgical repair, spinal cord deficit due to ischemia is a dreadful complication that comes with paraparesis/paraplegia in 6–40% of patients [1, 2].

In this setting, lactate releasing is the epiphenomenon of cellular suffering due to insufficient oxygen delivery. When spinal cord experiences an ischemic event, lactate production occurs as neurons switch on anaerobic metabolism. As literature reports, when blood brain barrier (BBB) is intact, subarachnoid lactate level depends on local production, then it is due to neuronal hypoxic suffering [3]. To prevent spinal cord injury cerebrospinal fluid (CSF) drainage, aimed to reduce intrathecal pressure to provide a better blood perfusion, is a technique now accepted by anesthesiologists and cardiovascular surgeons, although its effectiveness is controversial [4, 5].

During descending aorta surgery, we usually monitor the lactate trend in CSF, as this type of operation exposes the patient to the risk of spinal ischemia due to aortic clamping proximally to Adamkiewicz artery. 

Normal spinal fluid lactate concentration ranges from 0.6 to 3.1 mmol/L. Its level is age related and it can rise when spinal ischemic event occurs, also depending on the lasting of the low flow state [6, 7].

In our hospital, descending aorta surgical repair is performed without cardiopulmonary by-pass. Our experience on this kind of operation arises from a series of 8–10 cases per year. 

Out of 8 cases observed in the last year, we report two comparable cases of thoracic aorta repair with different neurological outcome that could have been predicted by intraoperative trend of lactate in subarachnoid fluid.

## 2. Case Report 1

A 61-year-old male patient (ASA 3; BMI 26.0 kg/mq body surface area) underwent general anesthesia for thoracic aorta aneurysmectomy. Preoperative blood test did not show any significant alteration but high level of serum creatinine (4.0 mg/dL) due to chronic renal failure (CRF) not needing of renal replacement therapy (RRT); electrocardiogram showed atrial flutter with 2 : 1–3 : 1 conduction (heart rate 55–65 bpm) as a consequence of a previous myocardial infarction surgically treated with coronaric-artery by-pass graft (CABG) three years before. Preoperative echocardiogram showed a good global kinesis (ejection fraction 65%; left ventricle end-diastolic volume 113 mL). Aneurysm maximum diameter was 7.5 cm.

Surgical approach consisted of thoraco-phreno-laparotomic incision, and before left emithorax opening, one lung ventilation (OLV) started and tidal volume (TV) was set to 6 mL/kg. After 90 minutes from the beginning; surgeon clamped aorta 3 cm below the isthmus. The clamping phase lasted 60 minutes.

During the operation i.v. administration of Nitrate (0.5 mcg/kg/min) was needed to assure safe values of blood pressure, particularly during clamping phase, and “to protect” coronary circulation.

Before aortic clamping, patient received N-acetyl-cysteine 2.4 g i.v. bolus, Methyl-Prednisolone 2 g i.v. bolus, and Mannitol 18% 100 ml, aiming to protect kidney and as free-radicals scavenging.

Global intraoperative fluid administration was 7200 mL including 10 units of red blood cells and 7 units of fresh frozen plasma; bleeding resulted in about 5000 mL and 2700 mL was readministered by a red cells storage device. Subarachnoid fluid withdrawal was 75 mL.

Postoperatively, he was admitted to ICU, awakened, and successfully extubated on the day after. Unfortunately he experienced paraplegia, although intrathecal pressure was ≤10 mmHg. CSF lactate concentration raised during operation and returned to normal level (3.2 mmol/L) within 12 hours, postoperatively. Magnetic resonance exam of spinal cord excluded local bleeding.

He was dismissed to ward after 6 days and left hospital 24 days after the operation.

## 3. Case Report 2

A 72-years-old male patient (ASA 3; BMI 23.1 kg/mq body surface area) underwent general anesthesia for thoracic aorta aneurysmectomy. Preoperative blood test did not show any significant alteration but Kaliemia 5.1 mmol/L and serum creatinine 1.29 mg/dL; electrocardiogram showed a sinus rhythm (68 bpm); echocardiogram reported a good global kinesis (ejection fraction 60%; left ventricle end-diastolic volume 78 ml). Eight years before, he experienced a myocardial infarction treated with percutaneous angioplasty and stenting. Aneurism maximum diameter was 6.7 cm.

Nitrate was not administered even during clamping phase; conversely, hemodynamics needed to be supported by Dopamine administration (8–10 mcg/kg/min).

Surgical approach consisted of thoraco-phreno-laparotomic incision, and before left emithorax opening, OLV started and TV lowered at 6 mL/kg. 

After 100 minutes from the beginning, surgeon clamped aorta 5 cm below the isthmus. The clamping phase lasted 105 minutes.

Before aortic clamping patient received N-acetyl-cysteine 2 g i.v. bolus, Methyl-Prednisolone 2 g i.v. bolus, and Mannitol 18% 100 ml, aiming to protect kidney and as free-radicals scavenging.

Global intraoperative fluid administration was 8500 mL including 4 units of red blood cells and 4 units of fresh frozen plasma; bleeding resulted in about 2000 mL and 625 mL was readministered by a red cells storage device. Subarachnoid fluid withdrawal was 100 mL.

Postoperatively, he was admitted to ICU, awakened, and successfully extubated on the day after. Patient did not experience any neurological postoperative complication and was dismissed to ward after 2 days and left hospital 15 days after the operation.

### 3.1. Common Features

In both of the two cases, aortic aneurysm involved the supradiaphragmatic tract of the vessel.

General anesthesia was induced with Fentanyl 1 mcg/kg i.v. + Propofol 2 mg/kg i.v. after preanesthesia administration of Morphine 5 mg + Atropine 0.5 mg i.m. Orotracheal intubation (double-lumen tube n.39) was performed after myorelaxation with cis-Atracurium 10 mg i.v. bolus and further administration of 0.15 mg/kg every 30 minutes. Anesthesia continued with administration of a gas mixture of air and oxygen (FiO_2_ 0.5-0.6, according to blood gas-analysis (BGA) results) and Sevoflurane 1-2%. Mechanical ventilation setting was tidal volume (TV) 5–8 mL/kg according to one-lung ventilation (OLV) or bilateral lung ventilation (BLV) phase; respiratory rate was 12 apm and it was modified to assure normal pCO_2_; positive end-expiratory pressure (PEEP) was 5–7 cmH_2_O. 

Intraoperative monitoring included electrocardiogram (D2 and V5) and ST line analysis, heart rate (HR), not-invasive blood pressure (NIBP) and invasive blood pressure (IBP) after right radial artery line insertion connected with FloTrac/Vigileo sensor (Edwards Lifesciences, Irvine, USA) for Cardiac Output (CO; Cardiac Index, CI) monitoring, peripheral oxygen saturation (SpO_2_), central venous pressure (CVP), end-tidal carbon dioxide (EtCO_2_), and diuresis. A subarachnoid catheter was inserted (Lumbar Drainage Catheter 46 cm, Codman and Shurtleff, Inc., Raynham, MA, USA) at lumbar level L3-L4 and connected to standard multichannel patient monitoring system to measure intrathecal pressure (*P*_Lq_) and withdraw CSF to lower it when necessary. Transesophageal echocardiogram (TEE) was used to value cardiac performance, particularly during the clamping period. BGA was tested after surgical incision, after OLV starting, every 15 minutes during aortic clamping, after unclamping, and after BLV restoring (Table 1). 

Postoperative analgesia started before the end of operation with i.v. infusion of a saline solution containing Morphine 30 mg/50 ml (2.1 mL/h). After the operation, the patient was sedated with Propofol 100 mg/h and admitted to ICU as planned to continue postoperative care and monitoring.

## 4. Discussion

Here are two case samples that we report to point out that liquor lactate level could be predictive of postoperative spinal cord damage due to ischemic event occurred during clamping phase of thoracic aorta surgical repair. 

**Figure 1:**
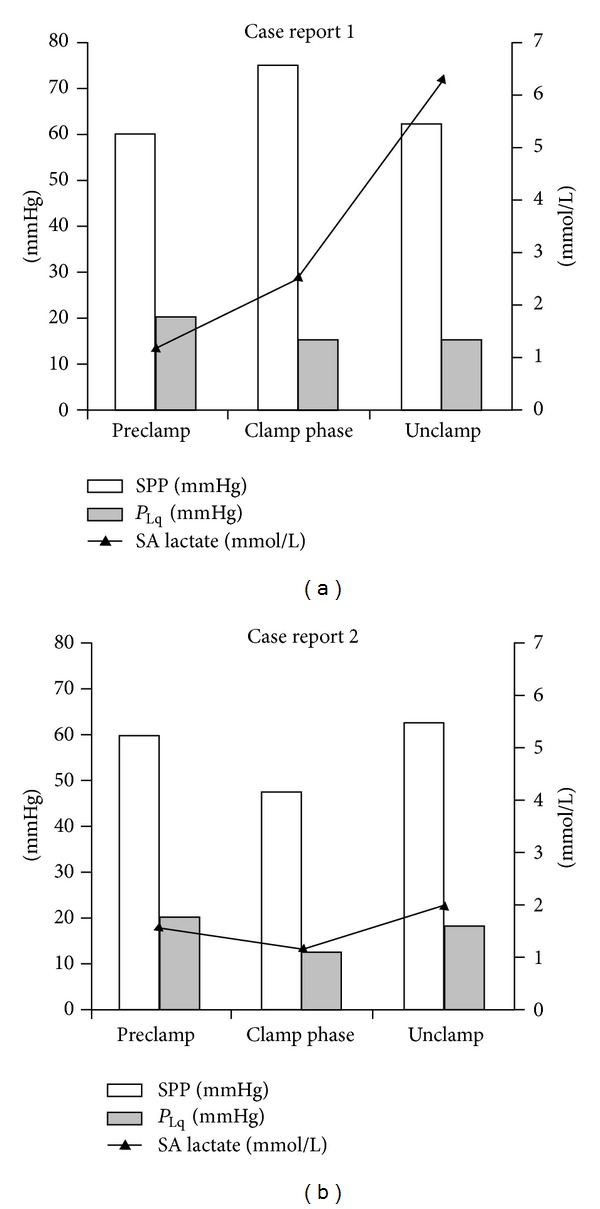
Subarachnoid pressure and Lactate. SPP = Spinal perfusion pressure (MAP-*P*_Lq_); *P*_Lq_ = intrathecal pressure; SA Lactate = lactate level in subarachnoid fluid.

Preoperatively, the two cases were very similar. They differed by the neurological outcome. The second patient did not experienced any complication, although he needed pharmacological hemodynamic support to provide an adequate blood flow. The former came out of operation with paraplegia. It happened although we lowered subarachnoid pressure significantly to assure a good spinal blood perfusion pressure (Figure 1). 

What may the cause be? Anatomical differences of vascular bed regarding Adamkiewicz artery origin, inadequate spinal perfusion pressure despite being in normal range, insufficient cerebrospinal fluid drainage could be some possible reasons of spinal suffering. Furthermore, another possible reason may be the different level at which aorta was clamped below the isthmus, although it consisted of only 2 cm. Anyway, whatever the cause, we expected a postoperative injured spinal cord in case 1 because, after the aortic clamping, subarachnoid lactate level rose progressively to a warning concentration.

As literature reports, when aorta is clamped subarachnoid pressure raises, probably due to a sympathetically mediated vasoconstriction that increases the tone of spinal veins with consequent venous engorgement [4]. Because of this issue, CSF withdrawal according to cerebrospinal fluid pressure is performed aiming to prevent spinal suffering from intrathecal hypertension.

Spinal cord low flow state can be detected by subarachnoid level of lactate production as it does not cross throughout blood brain barrier when it is not injured and then lactate blood-CSF mixing does not occur. It is a warning sign of neuronal anaerobic metabolism due to low flow state and its raising occurs earlier than other markers such as S-100 protein, [8]. 

The literature reports that CSF lactate level and other biochemical markers could raise without any postoperative neurological impairment [9]. We consider it may be only a possibility, not the rule. Since our habit is to monitor CSF lactate concentration, it was just the one case of postoperative paraplegia with significant intraoperative high level of subarachnoid fluid lactate concentration (>3 mmol/L).

Consequently, may CSF lactate level predict postoperative neurological outcome of spinal cord after descending aorta surgical repair? Several studies showed contrasting results and we are persuaded that a wide prospective trial should be performed aiming to answer the question.

In any case, CSF lactate concentration raising after descending aorta clamping should be a warning sign to make anesthesiologist do his best to prevent spinal cord ischemia/reperfusion injury even when all other parameters say that all is going well. Conversely, we consider that, whatever he does, it could be insufficient and postsurgical spinal damage is unavoidable.

## Figures and Tables

**Table 1:** Intraoperative data.

	Case report 1	Case report 2
Preclamping		
HR (bpm)	62	73
SpO_2_ (%)	98	100
MAP (mmHg)	80	80
CI (L/min/mq BSA)	2.4	2.7
SVV (%)	9	4
pH	7.33	7.35
pCO_2_ (mmHg)	40	38
p/F ratio	230	250
EtCO_2_ (mmHg)	25	29
Subarachnoid fluid withdrawal (mL)	40	60
*P*_Lq_/spinal perfusion pressure (mmHg)	20/60	20/60
Diuresis (mL)	300	500
Serum lactate (mmol/L)	2.2	1.7
Subarachnoid lactate (mmol/L)	1.2	1.6
During clamping		
HR (bpm)	80	62
SpO_2_ (%)	99	99
MAP (mmHg)	90	60
CI (L/min/mq BSA)	4.1	3.8
SVV (%)	4	5
pH	7.31	7.35
pCO_2_ (mmHg)	39	41
p/F ratio	165	154
EtCO_2_ (mmHg)	26	30
Subarachnoid fluid withdrawal (mL)	70	95
*P*_Lq_/spinal perfusion pressure (mmHg)	15/75	13/47
Diuresis (mL)	400	620
Serum lactate (mmol/L)	8.9	5.2
Subarachnoid lactate (mmol/L)	2.5	1.2
After unclamping		
HR (bpm)	83	86
SpO_2_ (%)	97	98
MAP (mmHg)	70	80
CI (L/min/mq BSA)	3.0	2.4
SVV (%)	7	14
pH	7.29	7.32
pCO_2_ (mmHg)	46	41
p/F ratio	208	240
EtCO_2_ (mmHg)	31	27
Subarachnoid fluid withdrawal (mL)	80	100
*P*_Lq_/spinal perfusion pressure (mmHg)	7/63	18/62
Diuresis (mL)	450	700
Serum lactate (mmol/L)	10.5	8.3
Subarachnoid lactate (mmol/L)	6.3	2.0

HR: heart rate; MAP: mean arterial pressure; BSA: body surface area; SVV: stroke volume variation; p/F: pO_2_/FiO_2_ ratio.
